# Magnetic tri-bead microrobot assisted near-infrared triggered combined photothermal and chemotherapy of cancer cells

**DOI:** 10.1038/s41598-021-87010-7

**Published:** 2021-04-12

**Authors:** Xiaoxia Song, Zhi Chen, Xue Zhang, Junfeng Xiong, Teng Jiang, Zihan Wang, Xinran Geng, U Kei Cheang

**Affiliations:** Department of Mechanical and Energy Engineering, Southern University of Science and Technology, Shenzhen, 518055 China

**Keywords:** Biomedical engineering, Mechanical engineering, Drug delivery, Cancer therapy, Targeted therapies

## Abstract

Magnetic micro/nanorobots attracted much attention in biomedical fields because of their precise movement, manipulation, and targeting abilities. However, there is a lack of research on intelligent micro/nanorobots with stimuli-responsive drug delivery mechanisms for cancer therapy. To address this issue, we developed a type of strong covalently bound tri-bead drug delivery microrobots with NIR photothermal response azobenzene molecules attached to their carboxylic surface groups. The tri-bead microrobots are magnetic and showed good cytocompatibility even when their concentration is up to 200 µg/mL. In vitro photothermal experiments demonstrated fast NIR-responsive photothermal property; the microrobots were heated to 50 °C in 4 min, which triggered a significant increase in drug release. Motion control of the microrobots inside a microchannel demonstrated the feasibility of targeted therapy on tumor cells. Finally, experiments with lung cancer cells demonstrated the effectiveness of targeted chemo-photothermal therapy and were validated by cell viability assays. These results indicated that tri-bead microrobots have excellent potential for targeted chemo-photothermal therapy for lung cancer cell treatment.

## Introduction

Cancer is one of the leading causes of death; lung cancer, in particular, is the biggest killer. To improve the effects of treatment, reduce the trauma caused by the operation, and overcome multidrug resistance, researchers developed stimuli-responsive drug release systems that can be triggered by external stimuli (such as magnetic^1^, electric^2^, and light^3^). Many studies have shown that photothermal therapy (PTT) with high spatiotemporal sensitivity can effectively kill cancer cells and avoid damage to normal tissues. Near-infrared (NIR) light can be converted into vibrational energy and rise the local temperature to induce tumor ablation^4,5^. Li et al. developed a type of benzo [c] thiophene (BT)-based photoactivated molecules to produce highly reactive oxygen species (ROS) when excited under near-infrared radiation (808 nm), which has an excellent anti-cancer effect on mice^6^. Ma et al. reported on NIR light-triggered molybdenum ditelluride nanosheets modified with targeting molecules and loaded with drugs for accurate diagnosis and treatment of tumor by combining photothermal and chemotherapy^7^. Although much progress has been made, the lack of precise and controllable targeting continues to hinder the development of targeted therapy using stimuli-responsive systems.

Magnetically controlled micro/nanorobots are actively investigated in recent years due to their promise to enhance the targeting ability of micro/nanoscale systems, such as drug delivery systems. Evidently, micro/nanorobots were applied in a wide range of fields, including military^8,9^, environmental^10,11^, and most especially biomedical applications^12,13^. Common biomedical applications with micro/nanorobots include cell transplant, targeted drug delivery for tumors, and minimally invasive surgery. To this day, many micro/nanorobots were successfully developed and demonstrated good motion control and precise targeting. Ceylan et al. fabricated a biodegradable double-helical microrobot for targeting drug delivery to SKBR3 breast cancer cells^14^. Kim et al. developed a magnetically actuated scaffold-type microrobot for targeted stem cell delivery to liver tumor tissue network^15^. However, given the complex and variable in vivo environments, the problems of poor flexibility of single shapes further limit micro/nanorobots in clinical application.

Recent research shows that magnetically controlled achiral robots possess excellent swimming abilities at low Reynolds number and are generally easy to fabricate due to their low geometric requirements. Inspired by particulate drug delivery systems (DDSs), Cheang et al. developed the tri-bead achiral microrobots with modular assembly^16^ and versatile motion control^17^. Using modular assembly and disassembly, the particle-based microrobots allowed for greater versatility to navigate different environments or perform multiple tasks. While the minimal geometric requirements and modulation are advantages of the achiral microrobots, there is no development towards using them for practical applications. Thus, there is a need to combine the advantages of achiral microrobots, the stimuli-responsive properties of smart drug release systems, and the targeting functions of magnetic microrobots in order to develop a feasible system for a precise and intelligent drug delivery system.

This work introduces a chemically assembled robust tri-bead anti-tumor microrobots with near-infrared triggered photothermal drug release. The tri-bead microrobots in this work are identical to the previously reported achiral microrobots with the same structural and magnetic property^18^; thus, ensuring these microrobots to possess the same excellent control and locomotive capability as the microrobots from previous work^16,17^. Since the motion control and mobility characteristics of the tri-bead microrobots were extensively studied in multiple previous reports, we only include a short video of a tri-bead microrobot under feedback control to demonstrate their mobility (see Video S1) and exclude a detailed characterization of their swimming properties. Thus, this microrobot combines the active propulsion mechanism of previously reported magnetic microrobots and stimulus-responsive chemo-photothermal treatment to enhance the effectiveness of targeted therapy. While Wang et al. reported a type of chemo-photothermal system, their microrobots were too large for selective targeting of cells and could only perform heat shock and burst drug release for 10 min due to the fast structural degradation of the microrobots in under 20 min; this essentially incapacitates the possibility for prolonged, sustained treatment on selective targets^19^. Here, NIR photo-responsive molecules (azo linker, 4,4′-azobis(4-cyanovaleric acid)) was used to link drug molecules onto the surface of tri-bead microrobots through covalent bonds, which provide excellent drug stability; upon NIR irradiation, the anti-cancer drug can be released and localized heat can be generated at the target position for a sustained period.

## Results and discussion

### Synthesis of tri-bead microrobots

**Figure 1:**
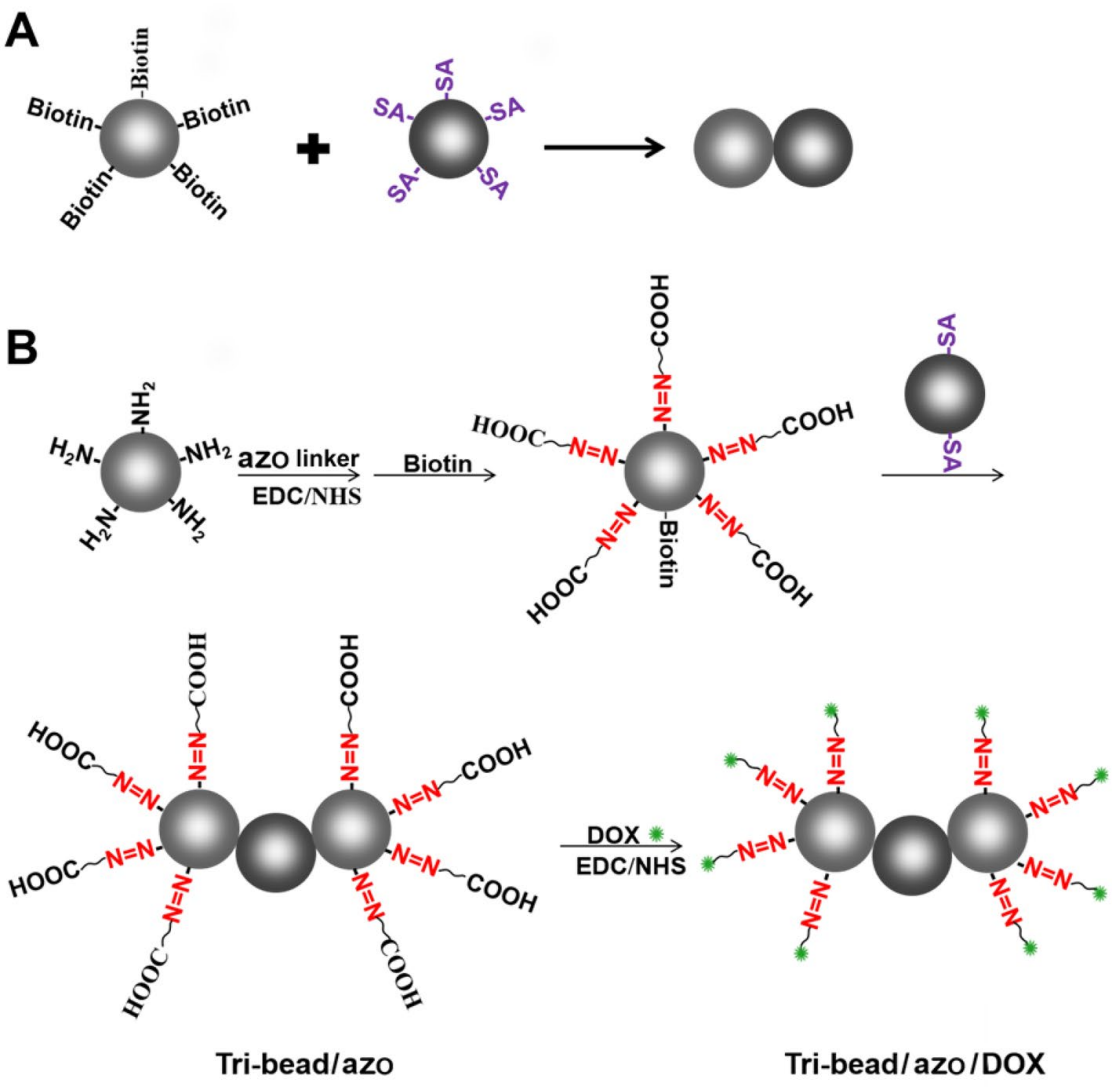
(**A**) The synthesis of bi-bead structures formed by conjugating biotin-coated magnetic beads with streptavidin (SA) coated magnetic beads. (**B**) Tri-bead microrobot linked by azo, and the chemical structure of azo and DOX.

**Figure 2:**
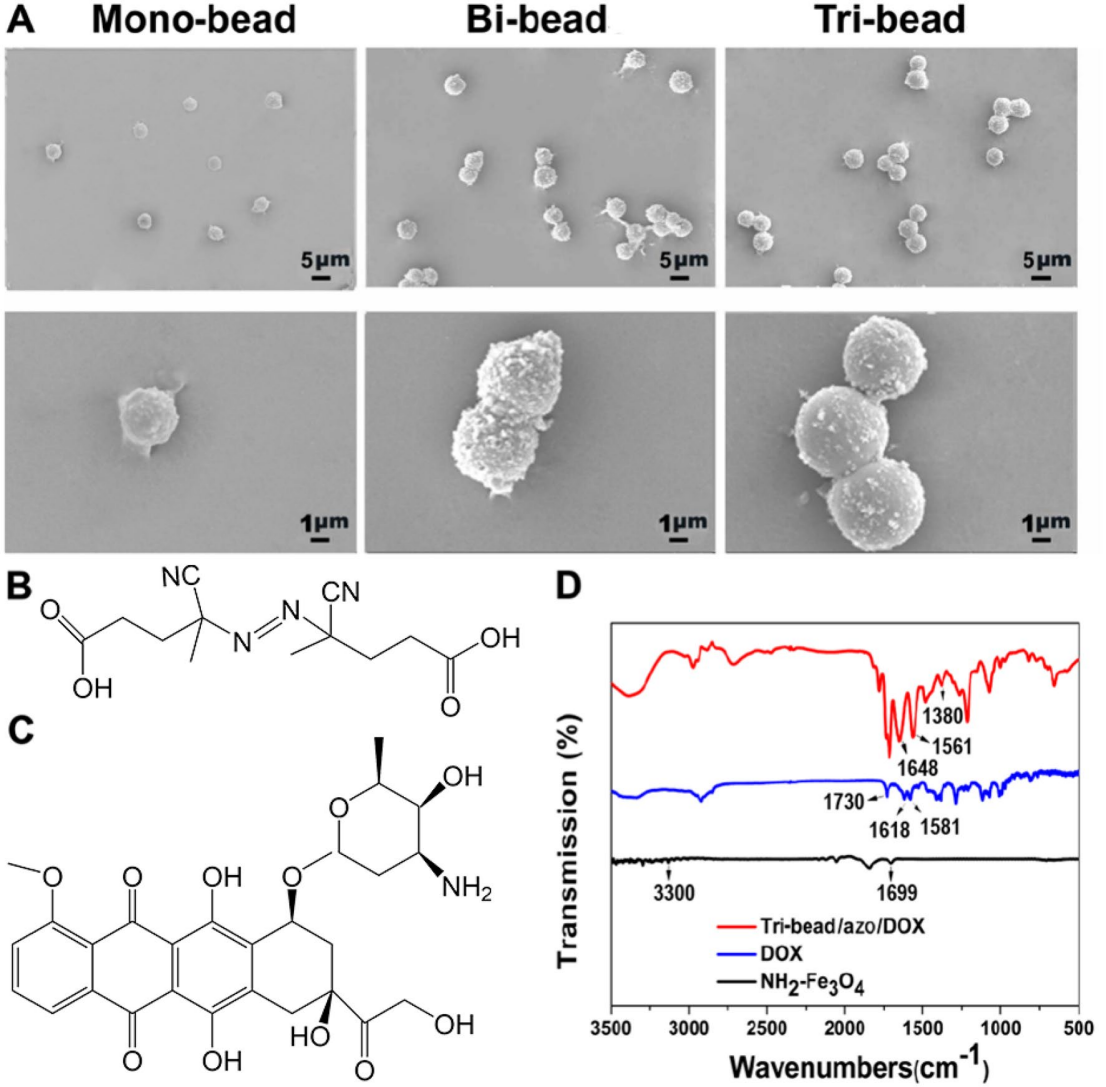
(**A**) The SEM morphology of the NH_2_-Fe_3_O_4_ microbeads, bi-bead structures, and tri-bead microrobots. (**B**–**C**) The chemical structure of azo and DOX. (**D**) FT-IR spectra of NH_2_-Fe_3_O_4_, DOX, and tri-bead/azo/DOX.

The schematics of bi-bead structures and tri-bead microrobots synthesis are shown in Fig. 1A, B. The microbeads used are 4.18 µm amino-modified magnetic microparticles (NH_2_-Fe_3_O_4_). The beads were evenly dispersed as single particles without aggregation (Fig. 2A). For preparing the tri-bead microrobots, first, dicarboxylic azo compounds and biotin were mixed with NH_2_-Fe_3_O_4_ microbeads to create azo/biotin beads. Then, azo/biotin beads are mixed with streptavidin-coated beads to create tri-bead/azo microrobots through stable streptavidin–biotin covalent binding. The number of biotin and streptavidin groups on the respective beads were kept low in order to reduce aggregation; as shown in Fig. 2A, the tri-bead microrobots (about 10 µm in length) can be obtained without aggregation and the number of three beads obtained by this method accounts for most of the proportion. Finally, DOX is added to the tri-bead microrobots to create tri-bead/azo/DOX microrobots; the N–H groups on the doxorubicin (DOX) can bind with the free -COOH groups on the azo linker. Bi-bead structures (7 μm in size) are also created using streptavidin–biotin interaction to serve as a comparison to the tri-bead microrobots (Fig. 2A). The chemical structure of azo and DOX are shown in Fig. 2B, C.

The successful loading of DOX on the microrobots was confirmed using Fourier transform infrared spectroscopy. As shown in Fig. 2D, the peaks around 1730 cm^−1^ belong to the C=O stretching vibration of C13 of DOX; more specifically, the peaks at 1618 cm^−1^ and 1581 cm^−1^ are assigned to the C=O stretching vibration modes of the anthracene ring (C6, C12)^20^. The spectra region (2800–3200 cm^−1^) is attributed to a combination of the stretching vibrations of the C-H, -OH, and N–H groups in the DOX. The peaks of 1699 and 3300 cm^−1^ are attributed to the amino-functional groups on the surface of Fe_3_O_4_ particles. The characteristic absorption peaks of azo at 1380 cm^−1^ (vCH_3_) and 1561 cm^−1^ (N=N) and the characteristic absorption peaks of DOX^15^ at 1648 and 1561 cm^−1^ were observed from the Fe_3_O_4_ particles modified with azo and DOX. These results indicate the successful loading of drug molecules using azo linkers. The drug loading efficiency (DLE) was calculated by measuring the concentration of DOX present in the supernatant by UV absorbance at 480 nm after centrifugation. We found the DLE of these microrobots to be around 5.3 μg/mg. The magnetic properties of the microrobots before and after drug loading were characterized using a vibrating sample magnetometer. As shown in figure S1, the microrobots are all superparamagnetic, and the saturation magnetization for tri-bead and tri-bead/azo/dox are 10.46 emu/g and 0.07 emu/g, respectively. A possible reason for such a dramatically low saturation magnetization for the drug-loaded robot sample is surface chemistry^21^.

### The photothermal effect of tri-bead microrobots

**Figure 3:**
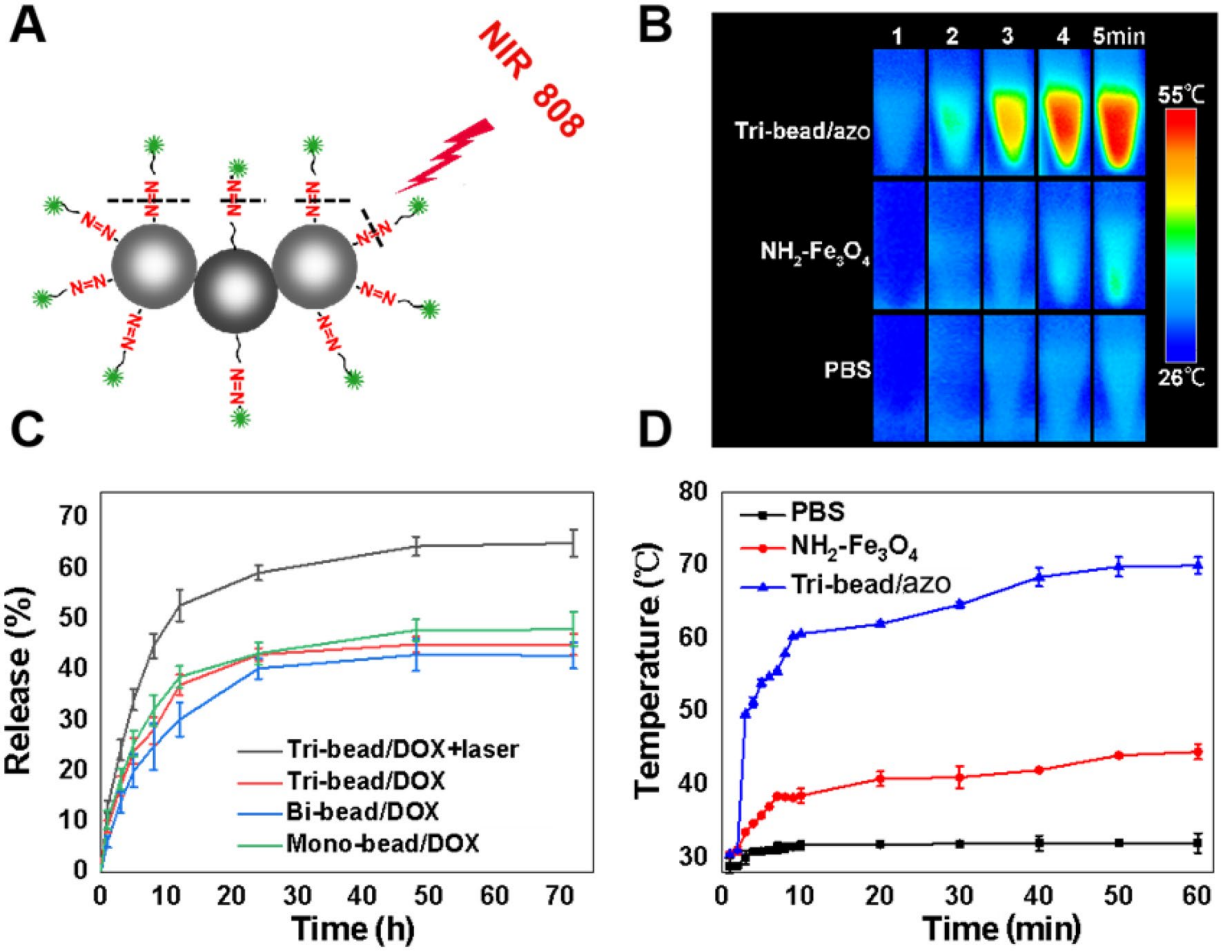
NIR-responsive drug release. (**A**) Schematic illustration of a tri-bead/azo/DOX microrobot irradiated by NIR light. (**B**) Thermographic images and (**C**) DOX release from tri-bead/DOX microrobots and NH_2_-Fe_3_O_4_ beads in PBS buffer at pH 7.4 with and without irradiation. Laser irradiation was performed for 5 min at different times. Asterisks indicate significant differences, *p < 0.05, and the sample number was n = 3. (**D**) Photothermal heating curves of tri-bead/azo microrobots, NH_2_-Fe_3_O_4_ beads, and PBS solution under 808 nm laser irradiation at a power density of 2 W/cm^2^ at different exposure time.

The azo group is a well-known thermosensitive molecule that has the function of optical switch drug release^22^ (Fig. 3A). The optical absorption spectra acquired with the tri-bead/azo/dox showed a broad absorption band spanning the UV and NIR regions (Figure S2). To evaluate the photothermal properties of tri-beads induced by azo, we compared the temperature change of PBS solution, PBS solution with NH_2_-Fe_3_O_4_ beads, and PBS solution with tri-bead/azo microrobot under NIR irradiation (808 nm, 2 W/cm^2^). As shown in Fig. 3B, the solution with tri-bead/azo microrobots shows a dramatic increase to 50 $^\circ$C at the center after four minutes, while the temperature change that was observed for the PBS solution and the solution with NH_2_-Fe_3_O_4_ beads were not significant and moderate respectively. As the irradiation time increased, the temperature of NH_2_-Fe_3_O_4_ and tri-bead/azo samples increase rapidly from 30.5 $^\circ$C to 38.5 $^\circ$C and 30.4 $^\circ$C to 60.6 $^\circ$C, respectively, within 10 min. After that, the rate of temperature increase slowed down and eventually reaching their respective maximum temperatures of 43 $^\circ$C and 70 $^\circ$C in 1 h (Fig. 3D). It should be noted that the temperature of 42 $^\circ$C and above can kill cancer cells^23^. Furthermore, the NIR exposure time can be controlled to avoid excessive heating. The results indicated that tri-bead/azo microrobots have outstanding photothermal conversion capability under NIR irradiation and can be used in photothermal therapy.

### NIR light-triggered DOX release from tri-bead microrobots

To detect the drug release triggered by NIR light, the samples were tested with and without NIR irradiation. As shown in Fig. 3C, while all of the samples follow a typical drug release curve with rapid drug release within the first 10 h and stable sustained release for the next 40–60 h, the case with tri-bead/azo/DOX microrobots with NIR irradiation shows a significant improvement. When NIR irradiation was applied to the tri-bead/azo/DOX microrobots, the DOX release was dramatically enhanced compared with tri-bead/azo/DOX microrobots without NIR irradiation; this validates the effectiveness of NIR triggered release. The cumulative release of DOX from tri-bead/DOX microrobots with NIR was 50% after 10 h and 60% after 30 h while that from the tri-bead/DOX microrobots without NIR was around 40% and 42%, respectively. Among the tri-bead, bi-bead, and mono-bead samples without laser irritation in Fig. 3C, the drug release profile did not show significant differences. The above results verify the good NIR light stimuli-responsive drug release performance of the tri-bead/azo/DOX microrobots.

### In vitro anticancer effects of tri-bead microrobots

**Figure 4:**
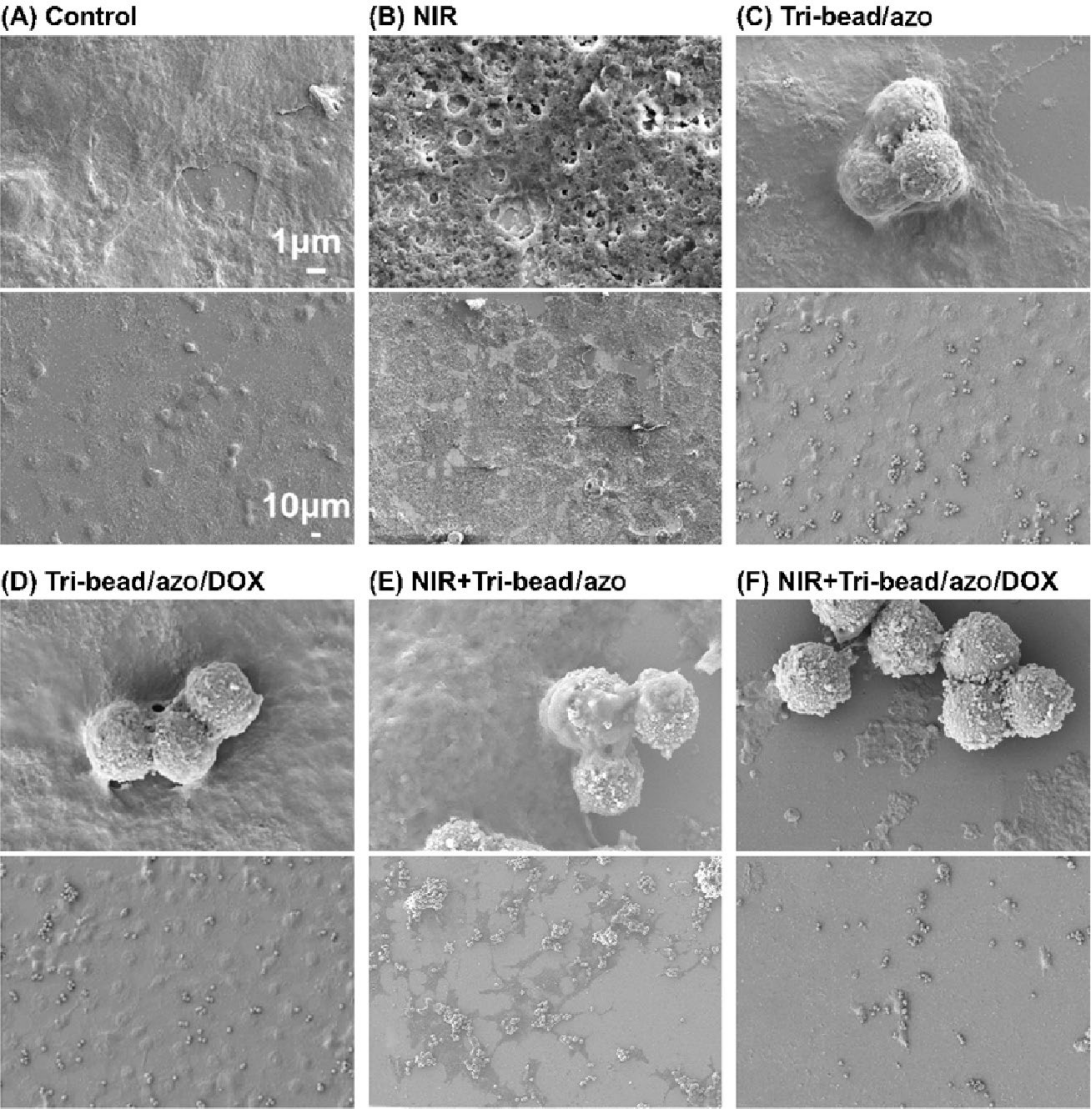
SEM images of H1299 cells incubated at different conditions: (**A**) control, (**B**) NIR irradiation, (**C**) tri-bead/azo microrobots, (**D**) tri-bead/azo/DOX, (**E**) tri-bead/azo with NIR irradiation for 24 h, and (**F**) tri-bead/azo/DOX with NIR irradiation for 24 h. The top and bottom images are zoomed-in and zoomed-out regions respectively.

To study the anticancer effects of the microrobots with NIR responsive drug release, a series of experiments were performed to compare six different groups- control with and without NIR, tri-bead/azo with and without NIR, and tri-bead/azo/DOX with and without NIR. Human lung carcinoma cells, H1299, were incubated for 24 h for each of the six cases and then fixed and dehydrated. The morphology of the H1299 cancer cells was characterized using SEM, as shown in Fig. 4. For the control and tri-bead/azo groups, there were no negative effects on cell viability; this is expected because high temperature and/or drug was not present, as shown in Fig. 4A,B. Considering that the laser itself can potentially damage the cancer cells, we studied the effects of 5 min of NIR irradiation with an 808 nm laser (2 W/cm^2^) on the cells; while there are observable changes to the surface morphology of the cells, there is no observable decrease in the number of cells compared to the control group (all areas of the substrate covered by cells), as shown in Fig. 4C. In contrast, tri-bead/azo with NIR irradiation significantly reduced the number of cells (large areas uncovered by cells) due to the high temperature from the photothermal effect, as shown in Fig. 4D. The comparison between Fig. 4C,D clearly shows the effectiveness of photothermal treatment of cells. To demonstrate chemo-photothermal treatment, we observed groups with tri-bead/azo/DOX with and without NIR irradiation. For the tri-bead/azo/DOX group without irradiation, there was no observable decrease in the number of cells and no changes to the cell morphology; this is because neither photothermal nor drug release took place. For the case with tri-bead/azo/DOX after NIR irradiation, cell number was significantly reduced compared to all of the other groups, and the cell morphology became observably abnormal compared to the tri-bead/azo group with NIR irradiation which indicates additional damages was done to the cells due to the NIR triggered the release of DOX. Furthermore, the CCK-8 assay was used to quantitatively evaluate cytotoxicity under different conditions (Figure S3). The results showed that 5 min of NIR irradiation was enough to trigger chemo-photothermal treatment on cancer cells and suggest that combined chemo-photothermal therapy is much more effective for cancer treatment than either modality alone.

**Figure 5:**
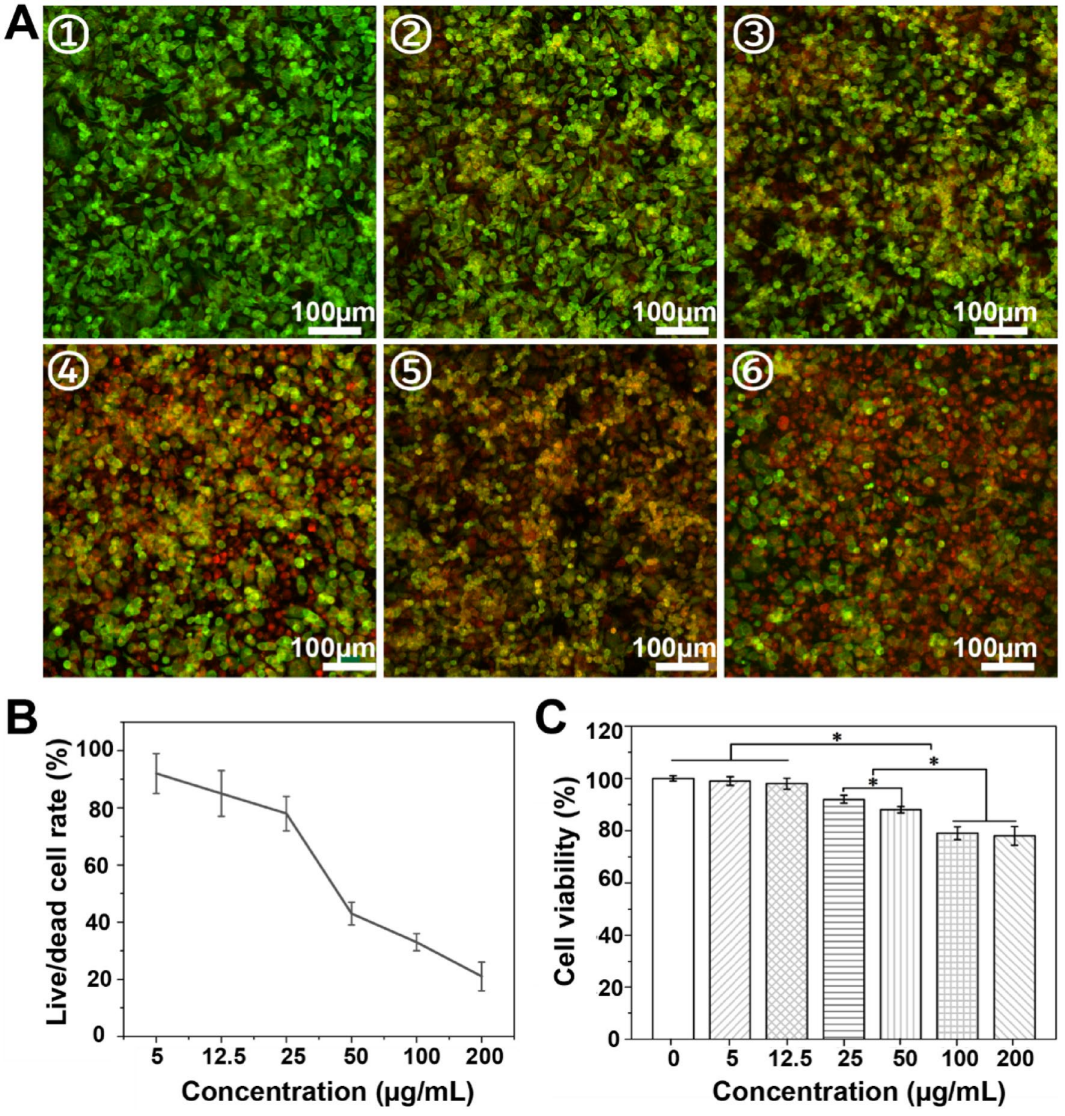
(**A**) Representative images of dead/live staining and (**B**) semi-quantification of the dead/live rate analysis with the ImageJ software. Live cells (green) were stained with calcein-AM, and dead cells (red) were stained with PI. Subfigures ①–⑥ show 5, 12.5, 25, 50, 100, and 200 μg/mL of tri-bead/azo/DOX microrobots with the NIR irradiation (2 W/cm^2^ for 5 min). The scale bar is 100 μm. (**C**) H1299 cells viability test by CCK-8 incubated with different concentrations of tri-bead/azo microrobots without NIR irradiation for 24 h. Asterisks indicate significant differences, *p < 0.05, n = 3.

Furthermore, the anti-cancer properties of tri-bead/azo/DOX microrobots were verified using live/dead staining assay, where the live cells and dead cells were stained by green (calcein-AM) and red (PI) dyes respectively. Cell viability decreased with increasing concentration of tri-bead/azo/DOX microrobots with NIR irradiation (2 W/cm^2^ for 5 min), as shown in Fig. 5A. The cell survival rate was semi-quantitatively evaluated and that only about 25% of the cells survived when the concentration of tri-bead/azo/DOX microrobots increases from 5 μg/mL to 200 μg /mL, as shown in Fig. 5B. Without NIR irradiation, the viability of H1299 cells, tested using Cell Counting Kit-8 (CCK8), was more than 75% even when the concentration of tri-bead/azo microrobots was up to 200 µg/mL, as shown in Fig. 5C; this is an indication that the tri-bead/azo/DOX microrobots without NIR-triggered heating and drug release cannot effectively kill the cancer cells. These results are consistent with the SEM images in Fig. 4 and demonstrate the anti-cancer effect of chemo-photothermal therapy.

### Magnetically guided targeted chemo-photothermal therapy in a microchannel

**Figure 6:**
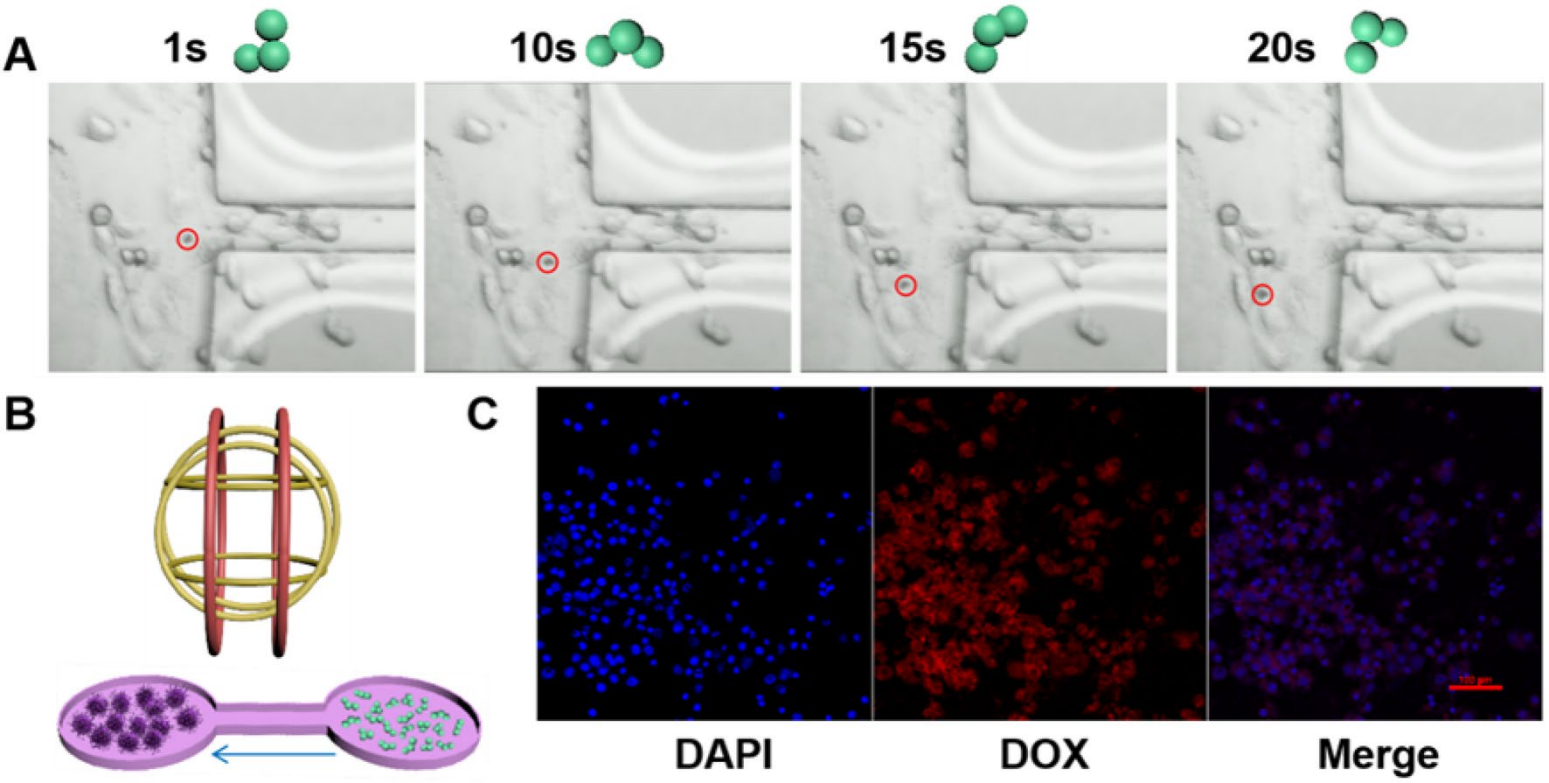
Experiments with targeted cancer cell treatment using magnetically actuated tri-bead/azo/DOX microrobots. (**A**) Movement position and swimming posture of a tri-bead microrobot at different times in the chamber with cancer cells. The Red circle indicates microrobot position. The rotation frequency of the actuation field is chosen as 5 Hz in the experiments. (**B**) Illustration of the magnetic control system and the experimental setup. The tri-bead/azo/DOX microrobots were placed in the right chamber and the H1299 cells were placed in the left chamber. (**C**) Cellular uptake tested performed using a confocal laser scanning microscope (CLSM), blue: DAPI; red: DOX.

To simulate targeted therapy in vitro, a bridge-type microchannel with left and right chambers was designed, as shown in Fig. 6B. The experiment was designed to test the feasibility to combine the mobility and targeting capability of the tri-bead microrobots with targeted anti-cancer treatment. Although the microchannel did not correspond to the physiology of a tumor site, it provides a controlled environment for experiments^24^. For the experiment, 20 μL of the H1299 cancer cells suspension (1 × 104/ml cell density) was added to the left chamber. The samples were dropped carefully into the chambers via pipetting to avoid overflowing and ensure the samples were separated. The cells were cultured for 1 to 2 h at 37 °C, 5% CO_2_ to allow the cells to adhere to the chamber. Then the fresh medium was supplemented for overnight culture. Then, the tri-bead/azo/DOX microrobots were transferred to the right chamber via careful pipetting. Next, the microrobots were magnetically controlled to move to the left chamber where the cancer cells are located. After reaching the cancer cell chamber, microrobots adhered to the cells upon contact and cannot move further. This is presumably due to the rough surface of cells and strong adhesion between the microrobots and the cells. Such interactions with the cells change the swimming postures of the microrobots, as shown by a representative microrobot in Fig. 6A (see Video S2); in other words, the change in posture is an indication that the microrobots came into contact with the cells. This phenohemon was observed in previous studies where the environmental conditions affected the motion of the microrobots^24^. The average velocity of the tri-bead/azo/DOX microrobots in this experiment was 14.5 µm/s. After the microrobots were magnetically guided to the cancer cell chamber, NIR laser irradiation (2 W/cm^2^) was carried out for 5 min. The cell uptake of DOX released from tri-bead/azo/DOX microrobots was confirmed using CLSM (Fig. 6C). The cancer cell nucleus was stained with DAPI. The intrinsic fluorescence property of drug DOX allowed for easy detection and identification through fluorescence microscopy. The staining results showed that in the case of NIR laser irradiation, DOX could be released from the tri-bead/azo/DOX microrobots near the nucleus of cancer cells. After the targeted drug delivery experiment, the cancer cells were cultured overnight. Further tests using CCK-8 showed that the survival rate of the cells was 50% (Figure S4). This indicates that NIR-triggered chemo-photothermal therapy led to excessive oxidative stress and ROS accumulation, which eventually leads to cell apoptosis. This result shows that the tri-bead/azo/DOX microrobots can successfully carry out magnetically guided targeted NIR-responsive chemo-photothermal therapy to cancer cells.

## Conclusion

In summary, NIR light-responsive drug-loaded robust magnetic tri-bead microrobots were fabricated and demonstrated targeted NIR-responsive chemo-photothermal therapy to cancer cells in in vitro experiments. We have proved that the tri-bead/azo/DOX microrobots can be synthesized in three steps. Chemical covalent bonds were used to connect the three magnetic beads as well as NIR-responsive linkers and drug molecules to the surface of beads; this provided much stronger and more stable bonds than physical adsorption. Drug release experiments showed that upon irradiation with near-infrared light (808 nm), tri-bead/azo/DOX microrobots were activated rapidly and caused the decomposition of azo molecules, which in turn released the drug molecules and increase the local temperature. The in vitro experiments showed that NIR-irradiated tri-bead/azo/DOX microrobots significantly inhibited the activity of H1299 cancer cells. Finally, magnetically guided targeted therapy performed in a microchannel demonstrated the tri-bead/azo/DOX microrobots can be magnetically guided to move to the cancer cell region, target specific cells, and perform NIR-triggered chemo-photothermal treatment on cancer cells. The results showed that the tri-bead/azo/DOX microrobots can provide potent synergistic chemo‐photothermal target therapy.

## Methods

### Materials

The magnetic microbeads were purchased from Spherobeads (USA). H1299 cancer cells were purchased from ATCC. RPMI 1640, fetal bovine serum (FBS), Trypsin–EDTA (0.25%), penicillin/streptomycin were purchased from Gibco (Gibco, USA). DAPI reagent (C0065) and anti-fluorescence quencher (S2100) were purchased from Solarbio (Beijing, China). CCK-8 assay was obtained from Dojindo (Japan). Calcein-AM/PI Double Stain Kit (C2015S) was from Biyuntian Biotechnology (Shanghai, China). Other reagents were purchased from Sigma unless otherwise noted.

### Preparation of Bi beads

The biotin-modified magnetic microbeads (4.45 µm, 1% w/v) were linked to the streptavidin-modified magnetic microbeads (4.10 µm, 1% w/v) to form bi-beads through biotin-streptavidin binding.

### Preparation of Tri-beads based microrobot

Tri-beads were fabricated in two steps. First, biotin (100 μg), 0.1 g of 4,4′-azobis (4-cyanovaleric acid) (azo) linker, and N-(3-dimethyl aminopropyl)-N′-ethyl carbodiimide hydrochloride (EDC, 0.035 g), N-Hydroxy succinimide (NHS, 0.028 g) were added to the amino-modified (NH_2_-Fe_3_O_4_) magnetic microbeads (100 μL, 4.18 μm, 2.5% w/v) solution. The mixture underwent shaking overnight at room temperature. The beads formed carboxyl groups and biotin on the surface. Second, streptavidin-modified magnetic microbeads were added to the beads from the first step to form tri-beads through biotin-streptavidin interaction. The tri-bead was washed three times with water and collected by freeze-drying. Infrared spectra were measured using the method of KBr disc with a PERKIN ELEMER Spectrum system. The morphology of magnetic particles was analyzed using SEM (Zeiss Merlin) and optical microscopy (MSHOT). Magnetic properties of the magnetic microrobots were measured using a vibrating sample magnetometer (VSM; Quantum Design PPMS DynaCool, LakeShore7404) at room temperature. The UV–vis–NIR absorption spectra were recorded on a Lambda25 spectrophotometer (PerkinElmer) with QS-grade quartz cuvettes at room temperature.

### DOX loading onto beads based microrobot

For DOX loading, 1 mg/ml DOX solution and EDC/NHS (1:1.2) dissolved in PBS buffer (0.01 M, pH = 7.4) were added to the bi- and tri-beads and mixed through vertexing for 24 h at room temperature. The drug-loaded beads were then collected by centrifugation and washed with water three times until the supernatant became clear. The drug concentration in the supernatant solution was measured by UV − Vis spectra at λ = 480 nm and calculated from a standard curve by measuring the absorbance of DOX solutions of known concentrations. The drug-loading efficiency (DLE) was calculated as follows: DLE (wt %) = weight of loaded drug/weight of total microrobots.

### Photothermal effects

Raw magnetic beads and tri-beads/AZO solution (1 mL) were irradiated under an 808 nm laser (2 W/cm^2^). At selected intervals (1 min), the real-time temperature change and thermal images of the samples were measured with an infrared thermal imaging system (Testo 865, Germany). PBS solution was used as the negative control.

### NIR-responsive dox release

DOX release from the beads/DOX samples was carried out by irradiating the 1.0 mg beads/DOX samples in 1 ml PBS solution (pH 7.4, 37 $^\circ$C) using an 808 nm NIR laser. After incubation for a period of time, 100 µl supernatant was taken out for detection, and an equal volume of fresh PBS was added into the test tube. The samples were irradiated for 5 min at different time points and analyzed by ultraviolet spectrophotometer (Infinite 200 pro, Tecan Austria GmbH, Austria) at 480 nm. DOX release was calculated from the slope of the standard curve. The experiment was performed 3 times.

### Cell culture

Human lung adenocarcinoma H1299 cells were used to evaluate the anti-cancer effect of the microrobot. Briefly, cells were cultured using R1640 supplemented with 10% fetal bovine serum (FBS) and 1% penicillin/streptomycin at 37 $^\circ$C, 5% CO_2_ in a humid environment. The samples were sterilized by 75% alcohol before cell seeding and then placed in 96-well tissue culture plates. Then, 100 µl of cell suspension with a density of 1 × 10^4^ cells/ml was seeded onto the samples.

### Cell viability and cell morphology

The cell viability Kit-8 was employed to quantitatively determine the cytotoxicity of the samples. After 24 h of culture, 10% CCK-8 solution in cell culture medium was added and incubated at 37 $^\circ$C for another 2 h. The OD values at 450 nm were read by a multifunctional full wavelength microplate reader (Infinite 200 pro, Tecan Austria GmbH, Austria). The morphology was observed by FE-SEM.

The live/dead staining assay was performed using a Calcein-AM/PI Double Stain Kit. Cells were seeded in 96-well cell culture plates at a density of 1 × 10^5^ cells per well. Then different samples were added to corresponding wells. After 24 h incubation, cells were washed twice with PBS. Afterward, 100 μL Calcein-AM/PI Double Stain detection working solution was added following the manufacturer's instructions. After 30 min incubated, cells were washed twice with PBS. Finally, the living cells stained with Calcein-AM (λ_ex_/λ_em_ = 490 nm/515 nm) and dead cells stained with PI (λ_ex_/λ_em_ = 535 nm/617 nm) were monitored by a confocal microscope. The numbers of live and dead cells were counted using Image J.

### In vitro cell uptake of the tri-beads

The cell uptake of the tri-beads microrobot was investigated by fluorescent staining. Fluorescence images were captured using a confocal laser-scanning microscope (Nikon A1R confocal). Briefly, after 24 h of culture, the H1299 cancer cells were washed with PBS three times and fixed with 4% paraformaldehyde for 15 min, and then stained with DAPI solution following the manufacturer’s instructions. Subsequently, the slices were washed with PBS five times and sealed with an anti-fluorescence attenuating sealer before scanned by a confocal microscope.

### Magnetic control and microchannel fabrication

To verify the motion of the microrobots, motion control experiments in a microfluidic channel were conducted using a magnetic control system with feedback control^25^. The magnetic control system is composed of three pairs of Helmholtz coils, a National Instruments data acquisition (NI DAQ) device, a camera, a microscope, and a host computer^25^.

The microfluidic channel is dumbbell-shaped with a width of 70 μm and a length of 5 mm. The channel was fabricated using conventional photolithography. The cell and microrobot areas located at opposite ends of the channel are both circular-shaped with the same diameter of 3 mm. After fabrication, the channels were cleaned through sonication in acetone, isopropanol, and deionized water, followed by plasma treatment. Lastly, the channels were sterilized by steam (1 bar, 120 °C) for 20 min.

## Supplementary Information

Supplementary Information 1.

Supplementary Video 1.

Supplementary Video 2.

## Data Availability

All data generated or analyzed during the present study are included in this published article.
