# Supplementary material for: Magnetic tri-bead microrobot assisted near-infrared triggered combined photothermal and chemotherapy of cancer cells
Source: Sci Rep. 2021 Apr 12;11:7907. doi: 10.1038/s41598-021-87010-7 (PMC8041914; doi:10.1038/s41598-021-87010-7)
Supplement: Supplementary file 1 — Supplementary Information 1. [file 41598_2021_87010_MOESM1_ESM.docx]

Supporting Information

Magnetic tri-beads microrobot assisted near-infrared triggered combined photothermal and chemotherapy of cancer cells

Xiaoxia Song, Zhi Chen, Xue Zhang, Junfeng Xiong, Teng Jiang, Zihan Wang, Xinran Geng, U Kei Cheang*

Department of Mechanical and Energy Engineering, Southern University of Science and Technology, Shenzhen 518055, China
E-mail: Cheanguk@sustech.edu.cn


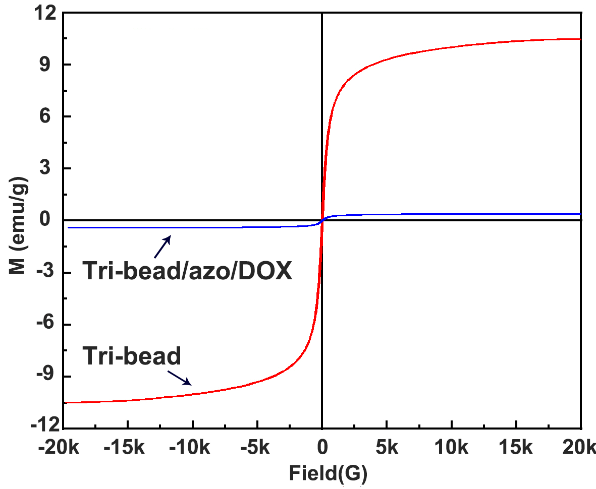


Figure S1. Magnetization curves of Tri-bead and Tri-bead/azo/DOX.

Figure S2. Absorbance spectra of AZO and Tri-bead/azo/DOX dispersed in water.


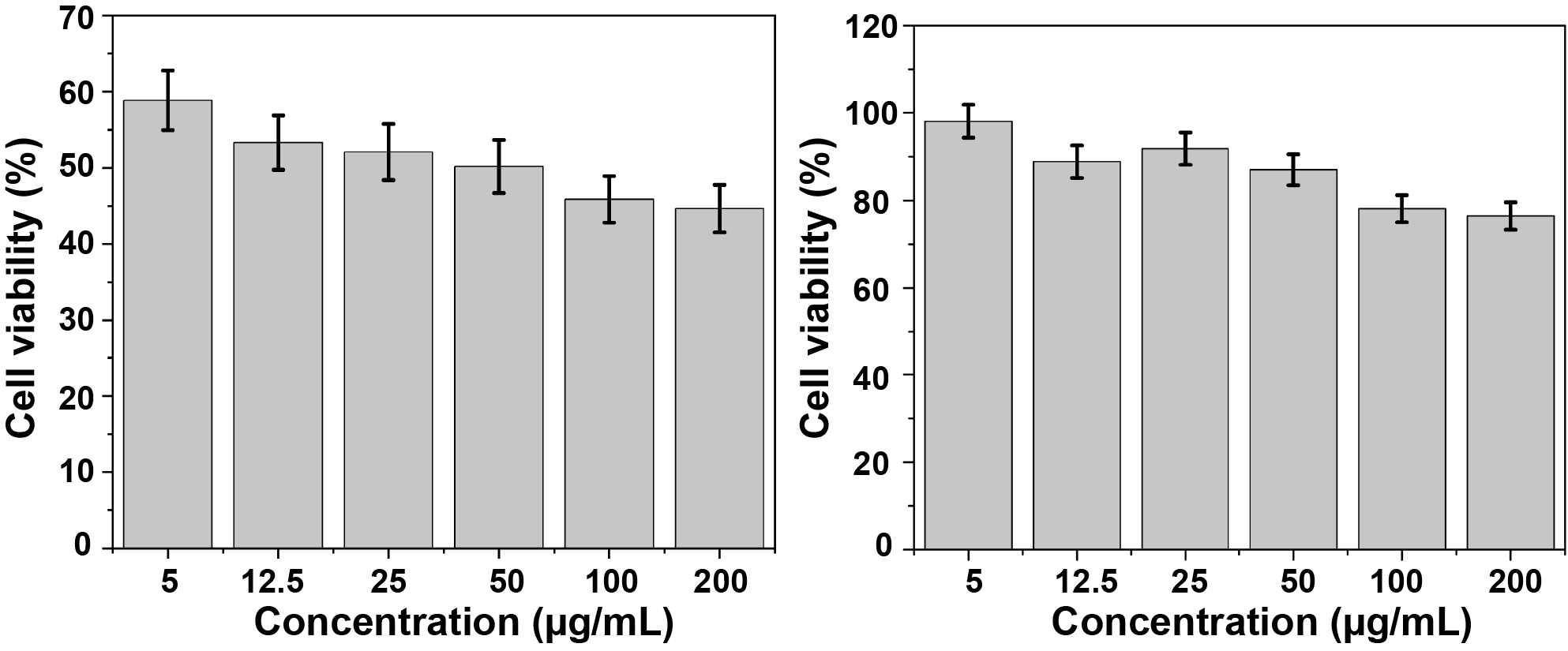


Figure S3. Cell viability of tri-bead/AZO with NIR irradiation and tri-bead/AZO/DOX.


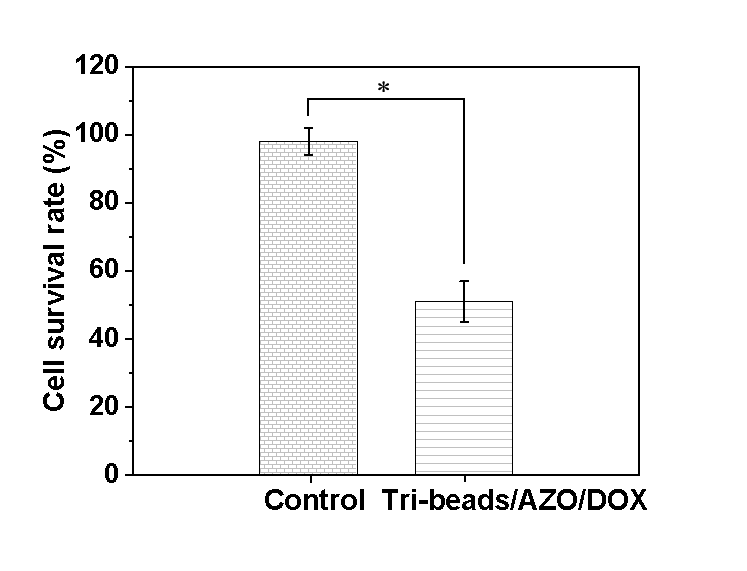


Figure S4. Cell viability of tri-bead/AZO/DOX under NIR irradiation after magnetic control in micro channel fabrication.
